# Effect of the Composition of Leuzea and Cranberry Meal Extracts on Metabolic Processes in Norm and Pathology

**DOI:** 10.3390/ph16050768

**Published:** 2023-05-19

**Authors:** Daria Khalikova, Sergey An’kov, Nataliya Zhukova, Tatyana Tolstikova, Sergey Popov, Anastasia Saiko

**Affiliations:** Vorozhtzov Institute of Organic Chemistry, Siberian Branch of the Russian Academy of Science, 9 Akademika Lavrentieva Ave., Novosibirsk 630090, Russia; sergey.ankov42@gmail.com (S.A.); gna2004@ngs.ru (N.Z.); tg_tolstikova@mail.ru (T.T.); spopov@nioch.nsc.ru (S.P.); sav@nioch.nsc.ru (A.S.)

**Keywords:** leuzea, cranberry, extracts, ursolic acid, ecdystene, adipocytes, metabolic syndrome, mice

## Abstract

This study was conducted to evaluate the effects of long-term administration of a new herbal composition of leuzea and cranberry meal extracts at a dose of 70:500 mg/kg in healthy and pathological mice. After 4 weeks of daily composition administration to healthy CD-1 mice and C57BL/6 mice with diet-induced metabolic syndrome, oral glucose tolerance test (OGTT), serum biochemical examination and histology of internal organs were performed. Additionally, histological examination of white and brown adipose tissue was performed to evaluate the ability of the composition to prevent abdominal obesity in C57BL/6Ay (agouti yellow) mice. The results showed that the composition increased tissue sensitivity to glucose in healthy CD-1 mice; at the same time, it did not worsen the course of pathological processes in pathological mice. In both cases, the application of the developed composition was safe and contributed to the restoration of metabolic parameters.

## 1. Introduction

The use of plant origin preparations is a current trend in modern medicine, pharmacology and cosmetology. Promising for these purposes are plant extracts with their constituent biologically active substances (polyphenols, flavonoids, triterpenes, phytohormones, glycosides, etc.), as they have a wide range of pharmacological properties, low toxicity and high availability in natural raw materials [1].

Phytoecdysteroids are the main biologically active compounds isolated from the roots, leaves and seeds of *Rhaponticum carthamoides* (commonly known as a maral root or leuzea) [2]. These are polar, steroidal, secondary metabolites, plant-produced analogues of ecdysteroids (insect moulting hormones), which control cell proliferation, growth and developmental cycles [3]. Known previously as ecdystene, β-ecdysone, or ecdysterone, 20-hydroxyecdysone is the most common and widely used biologically active compound of ecdysteroids with adaptogenic properties [4]. Nowadays, *Rhaponticum carthamoides* extract is included in numerous dietary supplements for its adaptogenic and tonic properties, antimicrobial, antioxidant, neuroprotective, antidiabetic, hypocholesterolemic and anabolic activities [5].

*Vaccinium oxycoccus* fruit is an exceptional source of biologically active compounds. Cranberry fruit peels contain significant amounts of ursolic acid, a pentacyclic triterpenoid of the ursanic type [6]. Ursolic acid has anabolic potential (enhances differentiation and mineralization of osteoblasts) and can promote skeletal muscle hypertrophy [7,8]. By increasing skeletal muscle mass and grip strength, ursolic acid promotes endurance (physical activity tolerance) [9]. Several works have shown that ursolic acid can also reduce fat gain and, thereby, prevent the development of abdominal obesity [10,11]. Ursolic acid has gained traction in the sports community because of its many potential health benefits as a potential phytochemical for functional nutrition.

A composition based on two plant sources consisting of leuzea and cranberry meal extracts containing 0.31% ecdystene and 40% ursolic acid, respectively, was developed in the Laboratory of Pharmacological Research NIOCH SB RAS (Novosibirsk, Russia).

The present study aimed to evaluate the safety of the composition of leuzea and cranberry meal extracts in long-term use and its effect on metabolic processes in healthy and pathological animals in vivo.

## 2. Results

### 2.1. Long-Term Composition Administration on Healthy Animals

During long-term (4 weeks) oral administration of the composition of leuzea and cranberry meal extracts at a dose of 70:500 mg/kg (the composition) to CD-1 mice of both sexes, no visual signs of intoxication were detected. Other physical parameters, such as local injuries, were also not identified. No animals died in all the groups during the experiment period. The body weight gain of the experimental animals (males, females) during the observation period was within the physiological norm. In addition, there were non-significant or no alterations in food and water consumption among the groups. There were no changes in fasting blood glucose concentrations from baseline.

These findings indicate that oral long-term administration of the composition had no effect on growth, feeding activity, and maintenance of body fluid volume in this experimental model.

**Figure 1:**
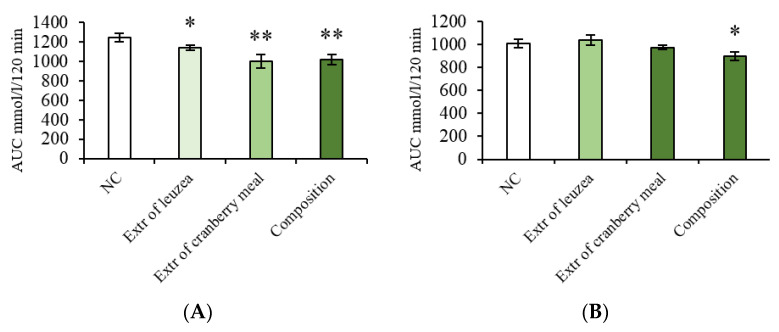
Area under the glycemic curve (AUC) calculated according to the oral glucose tolerance test (OGTT) data at the end of experiment, CD-1 mice. (**A**) Male; (**B**) Female. NC: negative control; Extr of leuzea: leuzea extract at a dose of 70 mg/kg; Extr of cranberry meal: cranberry meal extract at a dose of 500 mg/kg; the composition: composition of leuzea and cranberry meal extracts at a dose of 70:500 mg/kg. * *p* < 0.05 compared to Negative control; ** *p* < 0.005.

The results of the oral glucose tolerance test (OGTT) gave a histogram, where the value of the column corresponds to the area under the curve (AUC). AUC shows the total concentration of the administered drug (glucose) in plasma over the duration of the test. According to the test results, the composition promoted blood glucose utilisation in CD-1 mice of both sexes, significantly reducing its level compared to the negative control (NC) (Figure 1).

On macroscopic examination, the internal organs of the animals showed no irreversible, life-threatening pathology. The heart, liver, spleen, kidneys and organs of the reproductive system were not visually different from the control animals. There was no significant change in organ mass index between the study and control groups in both sexes.

Biochemical examination of the blood of CD-1 mice showed significant differences in terms of total protein (TP) in the leuzea extract at a dose of 70 mg/kg (Extr of leuzea), cranberry meal extract at a dose of 500 mg/kg (Extr of cranberry meal) and the composition groups compared to NC in both sexes. In males, the composition contributed to a reduction in lactate concentration relative to NC. Lactate levels in male mice treated by the composition were significantly decreased (Table 1).

### 2.2. Diet-Induced Models of Metabolic Syndrome

**Figure 2:**
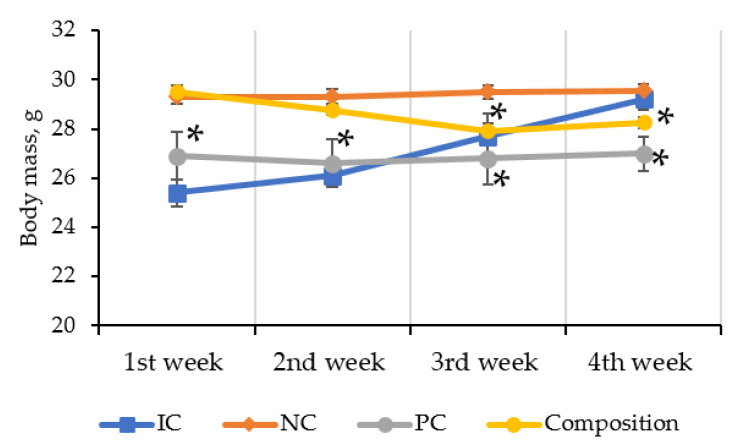
Body weight dynamics of C57BL/6 mice throughout the experiment (4 weeks). IC: intact control; NC: negative control; PC: positive control; Composition: composition of leuzea and cranberry meal extracts at a dose of 70:500 mg/kg. * *p* < 0.05 compared to negative control.

As C57BL/6 mice are known to be prone to obesity on a high-calorie diet (HCD), it was necessary to assess its effect on animal weight. There was a gradual, significant decrease in body weight of mice in positive control (PC) and the composition groups. However, there was no significant difference in the initial and final body weights of NC mice (Figure 2).

**Figure 3:**
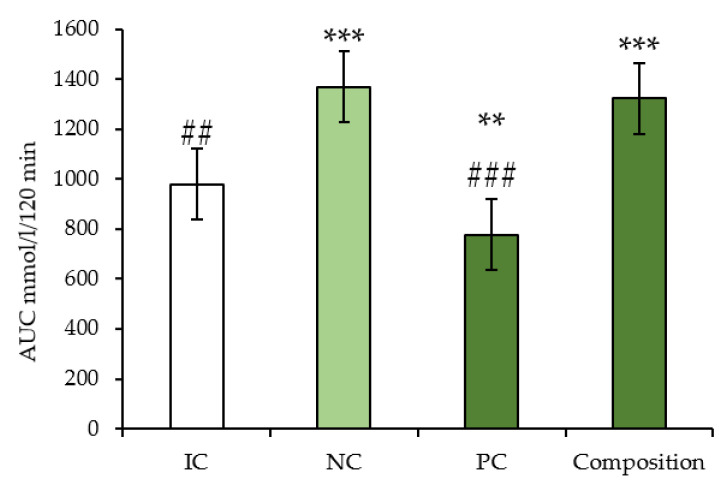
AUC calculated according to the OGTT data at the end of experiment, C57BL/6 mice. IC: intact control; NC: negative control; PC: positive control; Composition: composition of leuzea and cranberry meal extracts at a dose of 70:500 mg/kg. ** *p* < 0.005 compared to negative control; *** *p* < 0.0005. ## *p* < 0.005 compared to Intact control; ### *p* < 0.0005.

OGTT experiments were conducted to measure the effect of long-term (4th week) composition administration on blood glucose tolerance in C57BL/6 mice with metabolic syndrome. There was a characteristic impairment of glucose tolerance in NC and the composition groups. The drug metformin promoted a significant reduction in blood glucose levels (Figure 3).

During the biochemical blood assay, it was found that triglyceride (TG), high-density lipoprotein (HDL), AST (aspartate aminotransferase) and ALT (alanine aminotransferase) levels significantly reduced relative to NC. Total cholesterol (TC), TP, lactate, low-density lipoprotein (LDL), alkaline phosphatase (ALP) levels were the same as in IC (Table 2).

#### Histology

**Figure 4:**
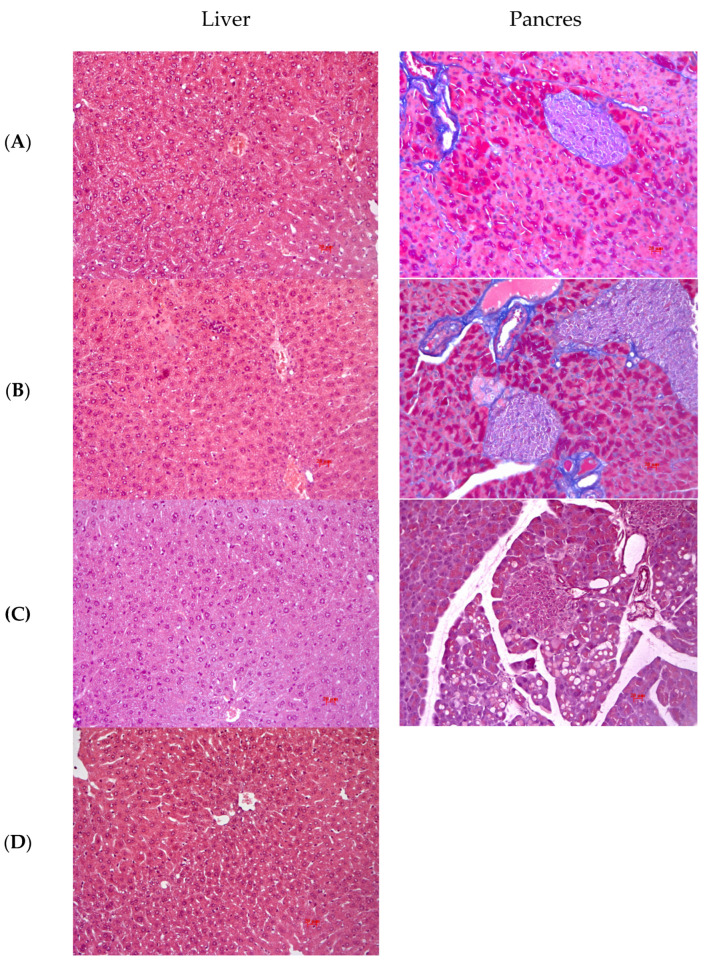
Histological evaluation of liver and pancreas in C57BL/6 mice at the end of experiment. (**A**) Intact control; (**B**) Negative control; (**C**) Composition: composition of leuzea and cranberry meal extracts at doses of 70:500 mg/kg; (**D**) Positive control: metformin at a dose of 250 mg/kg. Hematoxylin and eosin staining, ×200.

In intact animals, the liver architectonics was preserved; bile capillaries, veins and arteries had a typical structure; there were no signs of pathological infiltration, dystrophy and fibrosis. Figure 4A shows Kupffer cells without signs of increased activity. In the liver of NC mice, dystrophic changes in the form of small vesicular lipid infiltration were observed on venous profundity background. Numerous enlarged Kupffer cells were detected in the lumen of sinusoids (Figure 4B). Mice treated with metformin showed a decrease in the severity of dystrophic changes. Kupffer cells without signs of increased activity and mononuclear leukocytes were detected in the sinusoids (Figure 4D). In animals treated with the composition, degenerative and necrotic changes were detected, with small vesicular lipid infiltration in hepatocytes. A small number of enlarged Kupffer cells were noted in the sinusoids (Figure 4C).

The pancreas of IC animals is externally covered by a thin fibrous capsule, which is divided into lobules by the connective-tissue septa. The exocrine part is a complex tubular alveolar gland and occupies most of the organ. The endocrine part of the pancreas is formed by the islets of Langerhans; they have a well-developed network of blood vessels, which ensures an abundant blood supply to them (Figure 4A). In the exocrine part of the pancreas of NC animals, no pronounced alterations (dystrophy, necrosis, full thickness) were detected. Hyperplasia of islet apparatus was observed in the endocrine part. When stained by the Mallory tricolour method, several cells with bright purple staining were detected in the central part of the islet in individual animals. This is evidence that carbohydrate metabolism follows the pathway of gluconeogenesis, which is confirmed by the complete absence of glycogen in the liver (Figure 4B). Animals treated with the composition showed slight hypertrophy of the islet apparatus in the endocrine pancreas (Figure 4C).

### 2.3. Animal Model of Type II Diabetes Mellitus

Gradual weight gain was observed in both the study and NC groups, though not statistically significant during the study period.

White interscapular and gonadal fat masses in animals treated with the composition tended to be slightly reduced compared to the control group. There were no differences in the masses of interscapular brown fat (Table 3).

#### Histology

**Figure 5:**
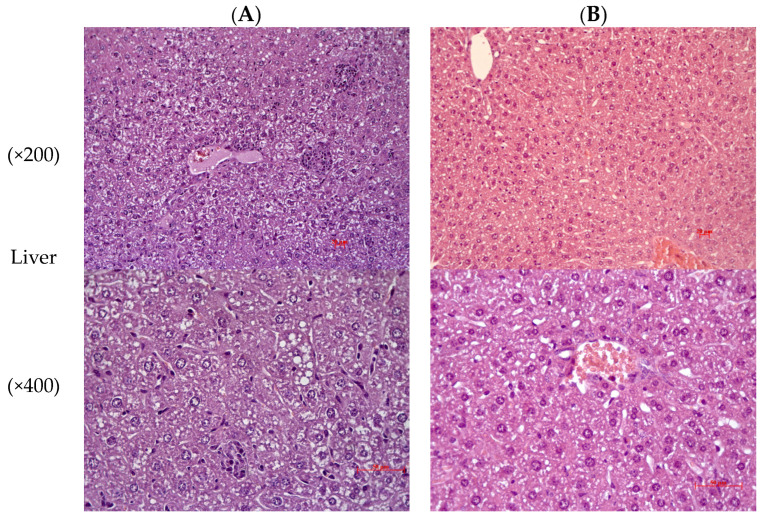

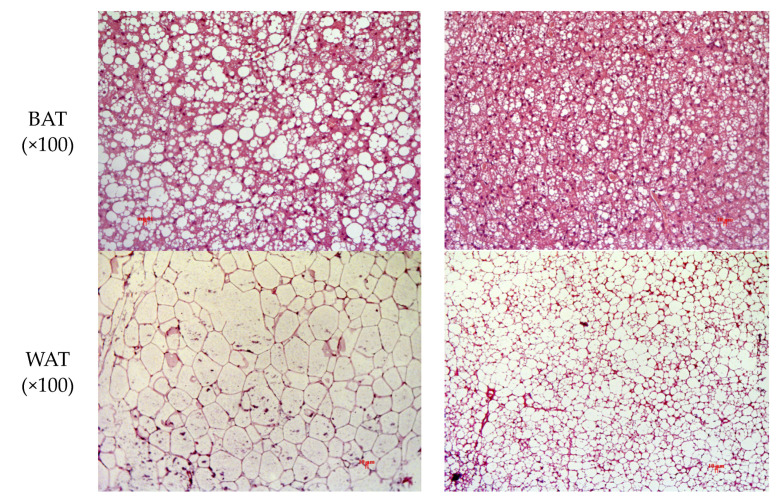
Histological evaluation of liver, brown adipose tissue (BAT), white adipose tissue (WAT) in C57BL/6Ay (AY) mice at the end of experiment. (**A**) Negative control; (**B**) Composition. Hematoxylin and eosin staining.

C57BL/6Ay mice NC showed signs of hepatocyte damage in the form of dystrophic changes in the liver, which were characterized by small vesicular lipid infiltration. In the parenchyma, there were single small focal necroses of hepatocytes in sinusoids activated Kupffer cells (increase in their size and number) (Figure 5A). The composition administration led to a reduction in the severity of dystrophic changes in the liver. Small vesicular lipid infiltration was detected in hepatocytes. No small focal necroses in the liver parenchyma were detected (Figure 5B).

The brown adipose tissue (BAT) of NC animals consisted of adipocytes containing a large number of varying size fat droplets, which fused together to form fat cysts. White adipose tissue (WAT) was formed by clusters of adipocytes of varying (mostly large) size, forming polygonal or rounded lobules separated by interlayers of connective tissue. The cytoplasm of adipocytes contained large drops of fat in the form of vacuoles. The flattened adipocyte nuclei were pushed to the cell periphery (Figure 5A). White and brown adipocytes of mice treated with the composition contained predominantly small fat droplets (decreased volume of lipid droplets). There were no signs of inflammatory changes in the adipose tissue (Figure 5B).

## 3. Discussion

The problem of increasing the biological and nutritional value of food, the rational use of raw materials, and the creation of new types of products with a wide range of pharmacological action is currently of paramount importance. The use of renewable natural compounds provides the possibility of obtaining additional products of high biological value and targeted use of natural resource reserves. For the development of plant composition, we used extracts for a more complete concentration of biologically active compounds (phytoecdysteroids and pentacyclic triterpenes). In our work, we used cranberry meal, which is a byproduct obtained in the process of fat extraction from the crushed seeds of various oilseeds, as a raw material for the production of cranberry extract. This will allow the most complete depletion of raw materials, recycling waste food industry [12]. An earlier study demonstrated a dose-dependent anabolic effect of the composition of leuzea and cranberry meal extracts at a dose of 70:500 mg/kg [13]. Another study demonstrated the ability of the composition to enhance physical performance [14]. However, there is evidence that the individual active ingredients (ecdystene and ursolic acid) have hypoglycaemic and hypocholesterolemic properties. In order to confirm the presence of these properties, it was necessary to study the effect of the composition on the lipid and carbohydrate profile in healthy and pathological mice. A diet-induced model of the metabolic syndrome and a model of animals with a genetic defect causing changes in the characteristic of the metabolic syndrome were used to simulate pathological conditions in animals.

According to the data obtained, long-term administration (4 weeks) of the composition to healthy CD-1 mice of both sexes had no effect on the physiological state of the animals, food and water consumption, body weight dynamics, and fasting glucose concentration. As a result of the OGTT, it was found that the composition at a dose of 70:500 mg/kg had the ability to reduce the concentration of blood glucose in CD-1 in both sexes. Biochemical analysis showed a significant decrease in total protein concentration in healthy mice of both sexes, which may be an indirect confirmation of the anabolic effect of the composition. Overall, there were no gender differences in the indicators examined.

In an experiment with a diet-induced model of metabolic syndrome, we used metformin as a positive control as a first-line drug for the treatment of type 2 diabetes mellitus. According to the literature, its effective dose is 200–250 mg/kg/d for mice, equivalent to a human metformin dose of 20 mg/kg/d [15]. After 4 weeks of daily administration of the composition, pathological C57BL/6 mice showed a decrease in body weight, which was also observed in the PC group. The OGTT results in the NC and the composition groups indicated a marked impairment of glucose tolerance. Thus, the composition had no hypoglycaemic effect in the pathological changes.

The main characteristics of dyslipidaemia are decreased HDL levels and increased TG and LDL levels. However, lipid (high-density lipoprotein) metabolism in mice and humans is not the same. Mice carry the majority of plasma cholesterol in HDL, whereas humans in LDL, causing the correlation of lipoprotein content in mice to be the opposite of that in humans [16]. Thus, an increase in HDL concentration for mice would be a negative factor. According to the results of a biochemical study, the composition significantly decreased HDL concentration of C57BL/6 mice. For TG, ALT and AST, it showed a similar effect to metformin, significantly reduced their concentration relative to NC. ALT and AST are mainly used to assess liver damage; elevated concentrations may indicate impaired metabolic function of the liver [17]. The values of other biochemical markers reflecting liver function (TC, LDL, ALP) didn’t differ from NC animals, which indicates the absence of hepatotoxicity. There were no significant differences in the parameters of protein and carbohydrate metabolism (TP, glucose, lactate).

The results of the histological examination indicated the presence of dystrophic changes in the liver (fatty hepatosis) and hyperplasia of the pancreatic islet apparatus in the NC group of C57BL/6 mice. An increase in the size and number of Kupffer cells indicated the presence of inflammation in the liver in this group [18]. In addition, areas with light-coloured beta cells were observed in the Langerhans islets, indicating the absence of insulin in them. The composition administration didn’t affect the development of fatty hepatosis, thus having no toxic effect on the liver. The endocrine part of the pancreas was hypertrophied but consisted predominantly of stained insulin-filled beta cells.

The effect of the composition on the redistribution of adipose tissue in an animal model of type II diabetes mellitus was studied in pathological C57BL/6Ay mice. With adequate nutrient intake, TG synthesised in the liver and intestine enter adipose tissue as lipoproteins and then hydrolyse to free fatty acids (FFAs). Subsequently, FFAs enter the vascular lumen and are taken up by adipocytes. There are two main types of adipocytes involved in the regulation of fat metabolism: white (WAT) and brown (BAT). White adipocytes are located in subcutaneous and visceral depots, the main function of which is the deposition of FFAs [19]. Brown adipocytes in small clusters are localised predominantly in the upper thoracic regions [20]. Unlike WAT, BAT does not perform the function of energy storage (in form of FFAs), but rather dissipates it through thermogenesis [21]. An increase in fatty deposits resulting from excessive food intake causes hypertrophy and hyperplasia of adipocytes through the accumulation of large numbers of lipid droplets inside the cells [22]. Thus, the size of adipocytes reflects the level of visceral fat in the body. The composition administration promoted the reduction of the size of white and brown fat adipocytes (i.e., the size of lipid droplets) in C57BL/6Ay mice, due to a tendency to decrease the mass of interlobular white fat. Histological examination of the liver in the animals group treated with the composition revealed a decrease in the severity of dystrophic changes. Thus, prolonged composition administration had no toxic effect on the structure of the studied organs.

## 4. Materials and Methods

### 4.1. Materials

1. Cranberry peel and seed meal extract containing 40% ursolic acid, obtained at the Engineering Centre NIOCH SB RAS (Novosibirsk, Russia) (File S4). Appearance: amorphous, water insoluble powder of light yellow to light brown color.
2. Aqueous-alcoholic radix leuzea extract containing 0.31% ecdystene, standardized by 20-hydroxyecdysone, was purchased from Altai Extracts Ltd. (Barnaul, Russia) (File S3). The substance is an amorphous, poorly water-soluble brown powder.
3. Composition of leuzea (0.31% ecdystene) and cranberry meal (40% ursolic acid) extracts.

The extracts were mixed mechanically and ground in a mortar with 2 drops of Tween 80, then distilled water was added.

### 4.2. HPLC Analysis

Since the compounds under study were purchased, it was necessary to confirm the quantitative content of the active substances in the extracts (File S1).

Chromatographic experiments were performed on a Milichrom A-02 microcolumn liquid chromatograph (EcoNova, Novosibirsk, Russia) with a column ProntoSIL-120-5-C18 (2 × 75 mm, 5 µm). In gradient elution mode, the mobile phase consisted of 0.05 M orthophosphoric acid (A) and acetonitrile (B). The mobile phase’s composition changed as follows: 0–7 min, isocratic condition, 10% B; 7–10 min, 10–55% B; 10–22.5 min, 55–100% B. Eluent flow rate of 0.2 mL/min, column temperature was set at 35 °C, analysis time 22.5 min, and sample volume was 8 μL. Wavelength for detecting ursolic acid in cranberry meal extract: 220 nm; for ecdystene in leuzea extract: 242 nm. Ursolic acid and ecdystene, as a standards marker, were used to analyze extracts. The arithmetic mean of three parallel determinations was taken as the result of the analysis, the allowed repeatability <5% rel.

### 4.3. MS Analysis

Mass spectra were recorded on a solution of 1 mg/cm^3^ of the sample in ethyl alcohol, direct input mass spectrometry (without chromatographic separation) on a Bruker micrOTOFQ instrument, electrospray ionization (ESI) method, negative ion registration mode, calibration mixture-arginine solution in acidified formic acid with bidistilled water (File S2).

### 4.4. Animals

The experiments were performed on 34 male C57BL/6 mice weighing 20–25 g, 80 CD-1 mice of both sexes (40 males and 40 females) weighing 25–30 g and 14 male C57BL/6Ay mice weighing 40–45 g. Animals were obtained from the vivarium of the Institute of Cytology and Genetics SB RAS.

Animals were kept under standard conditions in plastic cages (7–10 animals per cage) with wood shavings bedding under optimum conditions of temperature (21 °C ± 1.5), humidity, and 12/12 h light-and-dark cycle, with free access to food and water. All manipulations with animals were conducted in strict accordance with the laws and regulations of the Russian Federation, the decree of the Ministry of Health of the Russian Federation No. 199n of 4 January 2016, and the provisions of Directive 2010/63/EU of the European Parliament and of the Council of the European Union of 22 September 2010 on the protection of animals used for scientific purposes. The protocol of the animal experiment was approved by the Ethics Committee of N.N. Vorozhtsov Institute of Organic Chemistry SB RAS (protocol no. P-05-06.2022-14).

### 4.5. Long-Term Composition Administration on Healthy Animals

The physiological condition, body weight dynamics and macroscopic examination of internal organs were assessed in CD-1 mice of both sexes. Animals were randomly divided into four groups (*n* = 10), and the compounds tested were administered as follows: (1) “Negative control”: received the vehicle (distilled water + 2 drops of Tween 80); (2) “E-kt of leuzea”: received leuzea extract at a dose of 70 mg/kg; (3) “E-kt of cranberry meal”: received cranberry meal extract at a dose of 500 mg/kg; (4) “Composition”: received a composition of leuzea and cranberry meal extracts at a dose of 70:500 mg/kg. All compounds were given once a day by oral gavage for 4 weeks.

During the experiment, observational parameters were recorded daily. Body weight and fasting blood glucose levels were measured at the beginning of the experiment and at the end of each week. After four weeks of administration, OGTT was performed, after which the experiment was stopped, and animals were decapitated in order to collect blood for biochemical examination and the heart, liver, kidneys, spleen, testes and ovaries for macroscopic examination.

#### 4.5.1. Observational Parameters

The examination was carried out visually, and attention was paid to the following indicators: external covers (cleanliness, hair loss, abrasions, wounds, abscesses, growths), muzzle (moustache damage, eye inflammation), tail (clean tail cover, damage, deformities), behavior (mobility, aggressiveness), faeces (diarrhoea, no faeces), urine markings (color), feed and water (signs of wasting). The physiological condition was assessed daily for the next 4 weeks of the experiment.

#### 4.5.2. Fasting Blood Glucose

Blood glucose levels were measured using a glucometer ONE TOUCH Select blood glucose meter (LIFESCAN Inc., Milpitas, CA, USA). Blood was drawn from the tail vein of the animal by removing the tip of the tail with scissors, then a device with a test strip inserted was brought near and the glucometer automatically drew 1.5 µL of blood. The method of glucose determination is electrochemical, based on the biosensor glucose oxidase principle.

#### 4.5.3. Macroscopic Examination

At the end of the test, animals were weighed and then humanely sacrificed. Organs of interest, namely, the liver, heart, kidneys, spleen and suprarenal glands were carefully dissected out and weighed. Organ mass indexes (% of body weight) were calculated from the body weight data obtained.

### 4.6. Diet-Induced Models of Metabolic Syndrome

Male C57BL/6 mice were maintained for 6 months on a high calorie diet (HCD) with high fat (35.8%) and high carbohydrate (36.8%), consisting of a standard pelleted feed, which was mixed with butter and sugar. Pork fat was given in addition to the feed to accelerate the development of obesity and impaired glucose tolerance. After the development of impaired glucose and insulin tolerance (after 6 months), animals were randomised by weight and divided into 4 groups (*n* = 7–13): (1) “Intact control”: vehicle (distilled water + 2 drops of Tween 80) and standard pelleted feed; (2) “Negative control”: vehicle (distilled water + 2 drops of Tween 80) + HCD; (3) “Positive control”: metformin at a dose of 250 mg/kg + HCD; (4) “Composition”: composition of leuzea and cranberry meal extracts at a dose of 70:500 mg/kg + HCD. All compounds were given once a day by oral gavage for 4 weeks.

Body weight was recorded weekly, and an oral glucose tolerance test was performed after 4 weeks. At the end of experiment, mice were decapitated, blood was taken for biochemical examination, the liver and pancreas for histology.

### 4.7. Animal Model of Type II Diabetes Mellitus

Male C57BL/6Ay mice were used to study the composition effect on adipose tissue redistribution in an animal model of type II diabetes mellitus. These mice have several autosomal spontaneous yellow mutations at the agouti locus (which causes uncontrolled and ubiquitous expression of the Agouti gene) and, therefore, show different coat colouration, age-related obesity and insulin resistance [23].

Animals were randomly divided into four groups (*n* = 7), and the compounds tested were administered as follows: (1) “Negative control”: mice were given only vehicle (distilled water + 2 drops of Tween 80); (2) “Composition”: mice were given a composition of leuzea extracts and cranberry meal at a dose of 70:500 mg/kg. The studied compounds were given by oral gavage administration to their corresponding groups once a day continuously for 4 weeks.

Body weight was recorded weekly. At the end of experiment, the liver, white gonad fat, white and brown interstitial fat were quickly dissected out for histology.

### 4.8. Animal Body Weight Dynamics

The animals were weighed weekly throughout the experiment. The mice’s body weight was measured using a ViBRA CJ-620ER digital electronic balance.

### 4.9. Oral Glucose Tolerance Test (OGTT)

At the end of experiment, glucose levels were assessed against a glucose load. Mice were weighted and blood glucose was measured as a basal glucose value after fasting 12 h. All compounds were introduced 30 min prior to the glucose load by oral gavage. Then, mice were provided with glucose (2.5 g/kg, p.o.), and blood glucose was recorded at various time points (blood samples were obtained from tail incision) after injection (15, 30, 60, 90 and 120 min). A glycaemic curve was plotted on the basis of data obtained. AUC was calculated using Tai’s model [24].

### 4.10. Biochemical Assays

After the experiment period was over, the mice were sacrificed, the blood was centrifuged at 3000× *g* for 15 min to separate serum. The following parameters were determined: aspartate aminotransferase (AST), alanine aminotransferase (ALT), total cholesterol (TC), triglycerides (TG), total protein (TP), alkaline phosphatase (ALP), high-density lipoproteins (HDL), low-density lipoproteins (LDL), creatine kinase and lactate. Concentrations of these parameters were performed using standard diagnostic kits (Vector-Best, Novosibirsk, Russia) and a Stat Fax 3300 spectrophotometer (Awareness Technology Inc., Palm City, FL, USA).

### 4.11. Histological Examination

Histological analyses of the liver, pancreas, white gonad fat, white and brown interstitial fat of mice were performed by optical microscopy. Sections of the organs were fixed in buffered formalin (10%, *v*/*v*), processed by dehydration in different concentrations of ethanol, xylene and embedded in paraffin wax. Organs were sectioned 4 µm in thickness and stained with haematoxylin and eosin or Mallory tricolour for general examination of tissue structure. The slices were examined under a light microscope at a magnification of ×100–400.

### 4.12. Statistical Analysis

Statistical analysis was performed using Statistica 8.0 program. The data are presented in the format: mean ± standard error of the mean (SE). The normality of distributions was examined with the Shapiro-Wilk test. The studied variables displayed a normal distribution. Significance was assessed by the nonparametric Kruskal–Wallis test followed by the Mann–Whitney U test for the comparisons of independent groups within an experiment. A value of *p* < 0.05 was considered statistically significant.

## 5. Conclusions

There is a difference in the way the composition of leuzea and cranberry meal extracts at a dose of 70:500 mg/kg behaves in a healthy and pathological body. In a healthy body, this composition improves tissue sensitivity to glucose and increases total protein concentration (which mediates the anabolic effect). In pathology (diet-induced model of metabolic syndrome), it positively affects the biochemical parameters (ALT, AST, HDL, TG) and does not aggravate the course of pathological processes in the liver and pancreas. In mice with genetically determined obesity, it contributes to reducing the size of adipocytes of brown and white adipose tissue, thus preventing the development of hypertrophic obesity in mice. Accordingly, the application of the developed composition was safe and promoted the restoration of metabolic parameters. This makes the composition of leuzea and cranberry meal extracts promising for the development of biologically active supplements that contribute to the restoration and maintenance of metabolic processes in norm and pathology.

## Figures and Tables

**Table 1:** Blood biochemical parameters of CD-1 mice. NC: negative control; Extr of leuzea: extract at a dose of 70 mg/kg; Extr of cranberry meal: cranberry meal extract at a dose of 500 mg/kg; Composition: composition of leuzea and cranberry meal extracts at a dose of 70:500 mg/kg. TP—total protein.

	NC	Extr of Leuzea	Extr of Cranberry Meal	Composition
Lactate, mmol/L ♂	6.0 ± 0.07	5.8 ± 0.08	5.8 ± 0.11	5.7 ± 0.06 *
TP, g/dL ♂	8.7 ± 0.16	9.1 ± 0.12 *	9 ± 0.2	9.4 ± 0.3 *
TP, g/dL ♀	8.6 ± 0.15	9.4 ± 0.08 ***	9.4 ± 0.1 ***	9.2 ± 0.2 *

* *p* < 0.05 compared to Negative control; *** *p* < 0.0005.

**Table 2:** Blood biochemical parameters of C57BL/6 mice. IC: intact control; NC: negative control; PC: positive control; Composition: composition of leuzea and cranberry meal extracts at a dose of 70:500 mg/kg. TG—triglyceride, HDL—high-density lipoprotein, ALT—alanine aminotransferase, AST—aspartate aminotransferase.

	IC	NC	PC	Composition
TG, mmol/L	2.26 ± 0.02	2.33 ± 0.06	2.18 ± 0.02 *	2.20 ± 0.01 *
HDL, mmol/L	0.73 ± 0.01	0.73 ± 0.02	0.72 ± 0.01	0.68 ± 0.004 *
ALT, U/L	52 ± 3.63	50 ± 3.88	37 ± 2.85 *#	32 ± 1.99 *#
AST, U/L	159 ± 7.63	120 ± 4.12	98 ± 8.36 *	98 ± 11.06 *###

* *p* < 0.05 compared to Negative control. # *p* < 0.05 compared to Intact control; ### *p* < 0.0005.

**Table 3:** Mass of white gonadal fat, brown and white interscapular fat pads in C57BL/6Ay (AY) mice at the end of the experiment. NC: Negative control; Composition: composition of leuzea and cranberry meal extracts at doses of 70:500 mg/kg.

	NC	Composition
White gonadal fat, g	2.51 ± 0.18	2.39 ± 0.20
White interscapular fat, g	1.21 ± 0.09	1.13 ± 0.21
Brown interscapular fat, g	0.21 ± 0.02	0.19 ± 0.04

## Data Availability

Data is contained within the article.

## Supplementary Materials

The following supporting information can be downloaded at: https://www.mdpi.com/article/10.3390/ph16050768/s1. File S1. Description of HPLC analysis; File S2. Description of MS analysis; File S3. Quality passport for leuzea extract; File S4. Quality passport for cranberry meal extract.

## Abbreviation

ALP	alkaline phosphatase
ALT	alanine aminotransferase
AST	aspartate aminotransferase
AUC	area under the curve
Ay	agouti yellow
BAT	brown adipose tissue
Extr	extract
HCD	high-calorie diet
HDL	high-density lipoprotein
IC	intact control
FFAs	free fatty acids
LDL	low-density lipoprotein
NC	negative control
OGTT	oral glucose tolerance test
PC	positive control
TC	total cholesterol
TG	triglyceride
TP	total protein
WAT	white adipose tissue
