# Supplementary material for: Effect of the Composition of Leuzea and Cranberry Meal Extracts on Metabolic Processes in Norm and Pathology
Source: Pharmaceuticals (Basel). 2023 May 19;16(5):768. doi: 10.3390/ph16050768 (PMC10221024; doi:10.3390/ph16050768)
Supplement: Supplementary file 1 [file pharmaceuticals-16-00768-s001.zip › File S1.pdf]

HPLC analysis.

Chromatographic conditions:

Liquid chromatograph "Milichrom-A-02" (JSC "Econova", Novosibirsk)
Chromatographic column 75*2 mm filled with ProntoSIL-120-5-C18 sorbent, 5 µm
Eluent A: 0,05 M orthophosphoric acid (H3PO4), GOST 6552
Eluent B: Acetonitrile, TC 2636-092-444493179-04
Flow rate during regeneration and elution: 200 µl/min
Time of analysis: 22,5 min
Temperature: 35 °C
Detector: UV
Wavelength for Ursolic acid in cranberry meal: 220 nm
Wavelength for ecdystene detection in cranberry meal: 242 nm
Sample volume: 8 µl

Table S1. Gradient conditions

Step	V, µl	t, min	A, %	Б, %
Regeneration	600	3	90	10
Beginning	0	0	90	10
1	1400	7	90	10
2	2000	10	45	55
3	4500	22,5	0	100

Chromatographic experiments were performed on a Milichrom A-02 microcolumn liquid chromatograph (EcoNova, Novosibirsk, Russia) with a column packed with sorbent (2×75 mm, 5 µm, ProntoSIL-120-5-C18). Preparation of the chromatograph for the analysis was performed in accordance with the instrument operating instructions. Immediately before analysis, the eluent was degassed by barbotaging with helium. In gradient elution mode, the mobile phase consisted of 0.05M orthophosphoric acid (A) and acetonitrile (B). The mobile phase's composition changed as follows: 0–7 min, isocratic condition, 10% B; 7–10 min, 10–55% B; 10–22.5 min, 55–100% B. Eluent flow rate of 0.2 mL/min, column temperature was set at 35°C, and analysis time 22.5 min were used. Wavelength for detecting ursolic acid in cranberry meal extract: 220 nm; for ecdystene in leuzea extract: 242 nm.

The sample extract weighing 10 mg (with an accuracy of 0.01 mg) was dissolved in 10 ml of ethyl alcohol and centrifuged. The liquid part was used as a sample for analysis (approximate sample concentration 1 mg/ml). Using an autosampler, 8 µl of the sample solution was injected successively. Compounds were identified by comparing their UV spectra and retention times with those obtained from available standards.

The mass fraction of the main substance (ω, %) was calculated by the formula:

$$\omega \quad = \frac{S \quad \cdot C \quad \cdot P}{S \quad \cdot C} \cdot 100\%$$

where P – content of the main substance in the calibration sample (in fractions);

S – areas of active ingredient peak in the sample of extract and in the calibration sample;

C – concentrations of extract sample and calibration sample of active ingredient in the prepared solutions.

The arithmetic mean of two parallel determinations was taken as the result of the analysis, the allowed discrepancy between them should not exceed 5% r.t.
