# Supplementary material for: Effect of the Composition of Leuzea and Cranberry Meal Extracts on Metabolic Processes in Norm and Pathology
Source: Pharmaceuticals (Basel). 2023 May 19;16(5):768. doi: 10.3390/ph16050768 (PMC10221024; doi:10.3390/ph16050768)
Supplement: Supplementary file 1 [file pharmaceuticals-16-00768-s001.zip › File S2.pdf]

1. The first group of samples - ursolic acid and cranberry meal

1.1 Ursolic acid sample C30H48O3 (anion mass C30H47O3- 455.353)

Mass spectra were recorded on a solution of 1 mg/cm3 of the sample in ethyl alcohol, direct input mass spectrometry (without chromatographic separation) on a Bruker mi-crOTOFQ instrument, electrospray ionization (ESI) method, negative ion registration mode, calibration mixture - arginine solution in acidified formic acid with bidistilled water.

General view of the mass spectrum in the range 150-800 daltons:

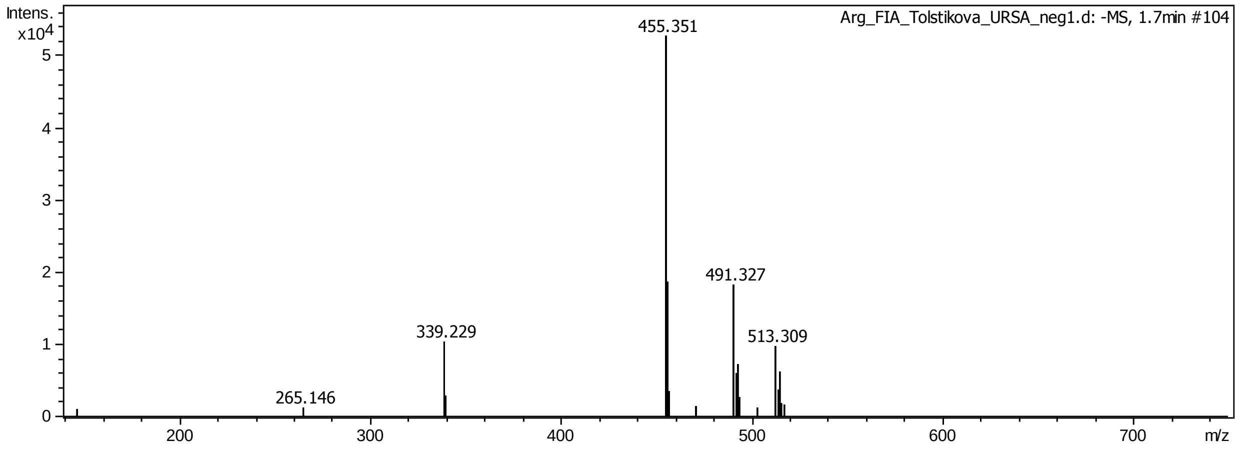

We can see that the negative ions have a peak with m/z=455 - this is the expected peak of the anion C30H47O3- and, additionally, there are isotopic peaks of the cluster.

The mass spectrum is around 455 daltons:

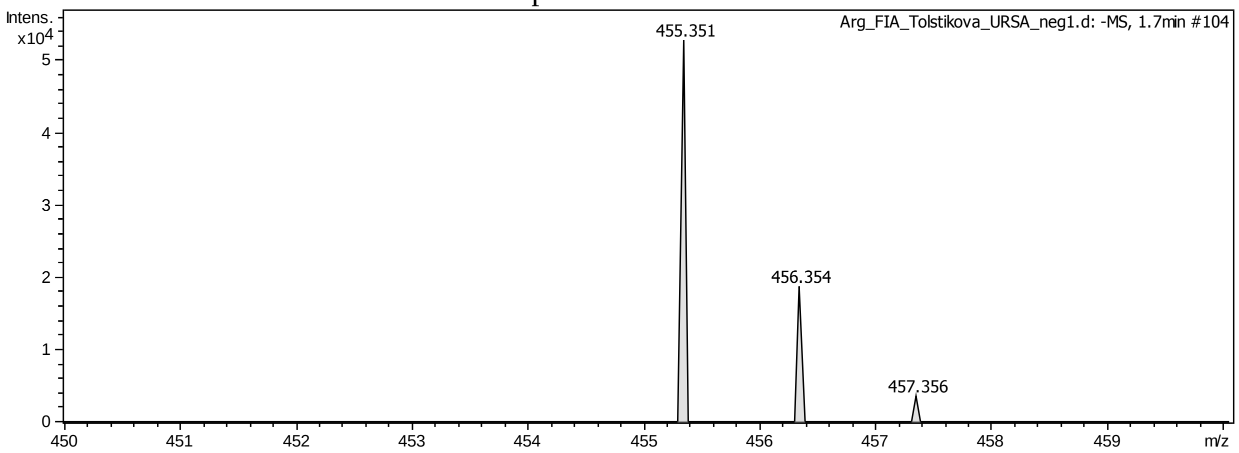

Simulation with C30H47O3-, m/z=455, recorded mass spectrum above, simulation below:

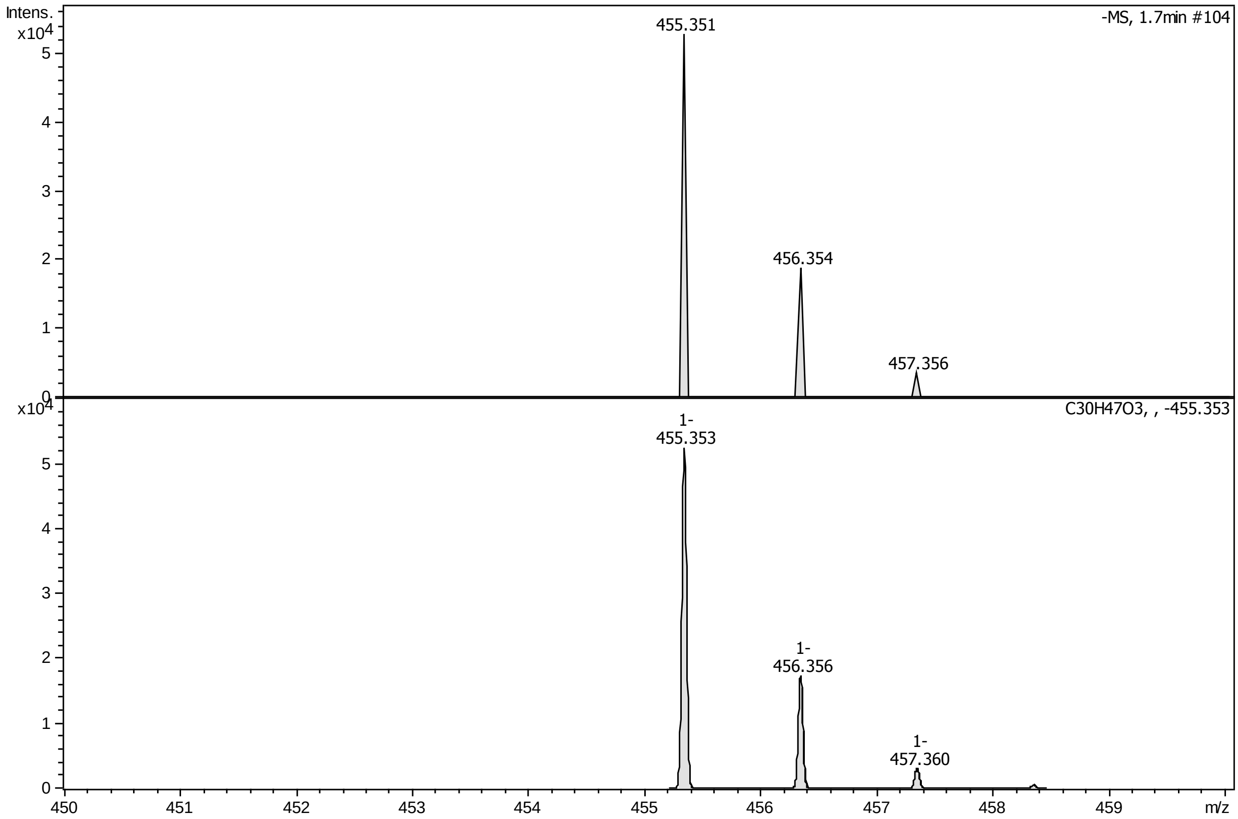

Thus: with an error of 0.002 daltons (4.4 m.d.) this is the structure, according to the isotopic distribution also looks like it.

1.2. Cranberry meal sample

Mass spectra were recorded on a solution of 1 mg/cm3 of the sample in ethyl alcohol, direct input mass spectrometry (without chromatographic separation) on a Bruker mi-crOTOFQ instrument, electrospray ionization (ESI) method, negative ion registration mode, calibration mixture - arginine solution in acidified formic acid with bidistilled water.

General view of the mass spectrum in the 300-750 daltons range:

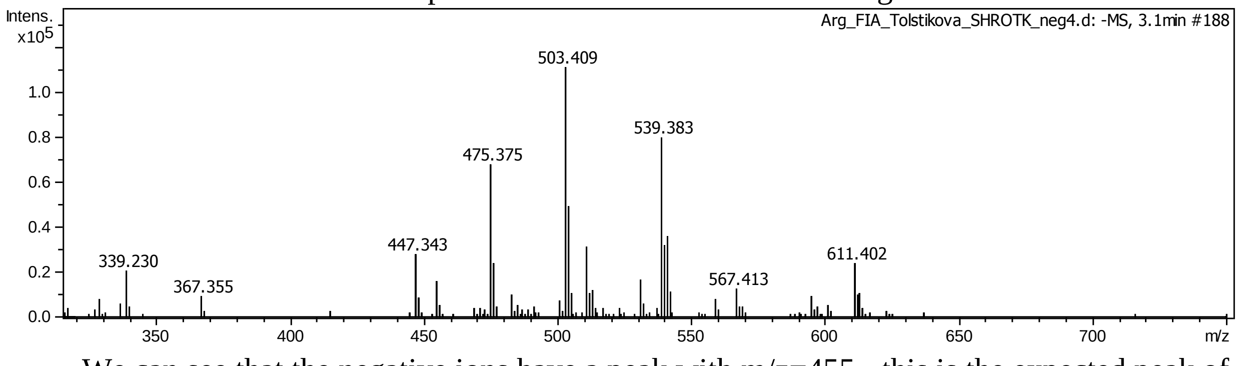

We can see that the negative ions have a peak with m/z=455 - this is the expected peak of the anion C30H47O3- and, additionally, there are isotopic peaks of the cluster.

Mass spectrum around 455 daltons:

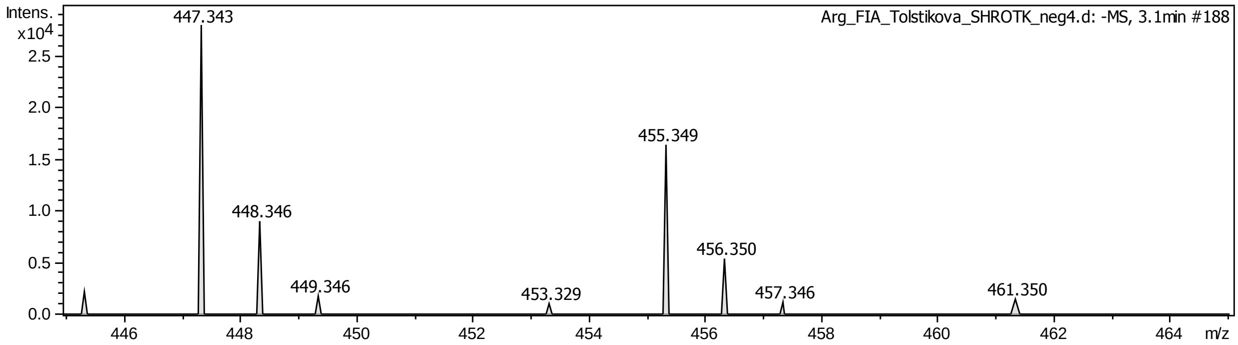

Simulation with C30H47O3-, m/z=455, recorded mass spectrum above, simulation below:

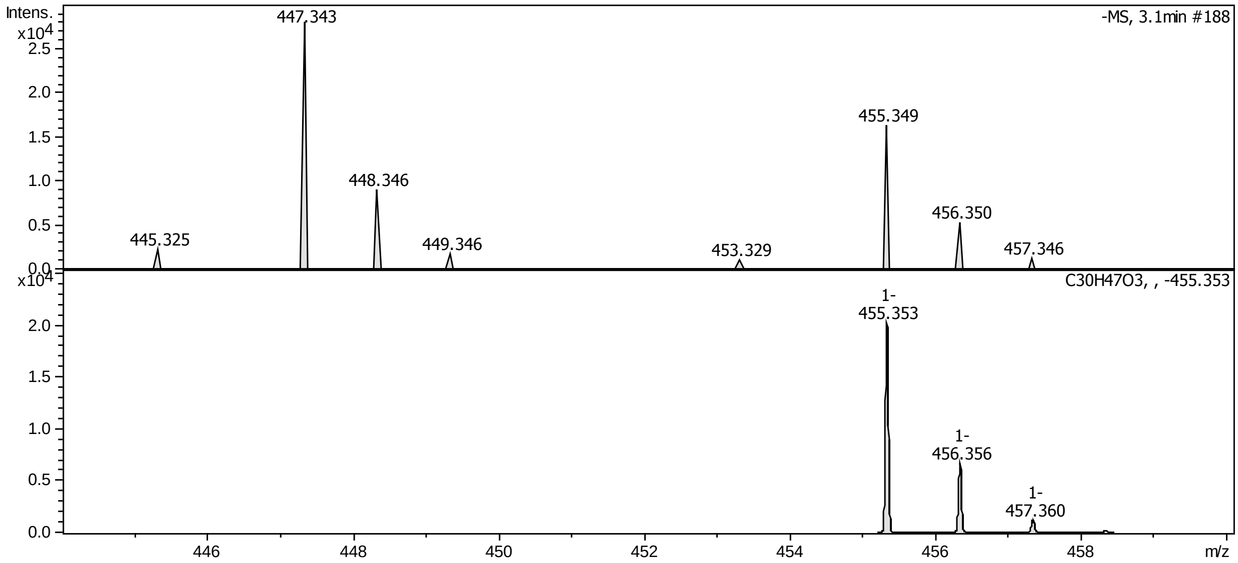

So: with an error of about 0.004 daltons (8.8 m.d.) this is the structure, according to the isotopic distribution also looks like it.

2. The second group of samples - ecdystene and leuzea extract

2.1 Sample of ecdystene $C_{27}H_{44}O_7$ (cation mass $(M+H)^+$ $C_{27}H_{45}O_7^+$ 481.317)

Mass spectra were recorded on a solution of 1 mg/cm3 of the sample in ethyl alcohol, direct input mass spectrometry (without chromatographic separation) on a Bruker mi-crOTOFQ instrument, electrospray ionization (ESI) method, negative ion registration mode, calibration mixture - arginine solution in acidified formic acid with bidistilled water.

General view of the mass spectrum in the 300-900 daltons range:

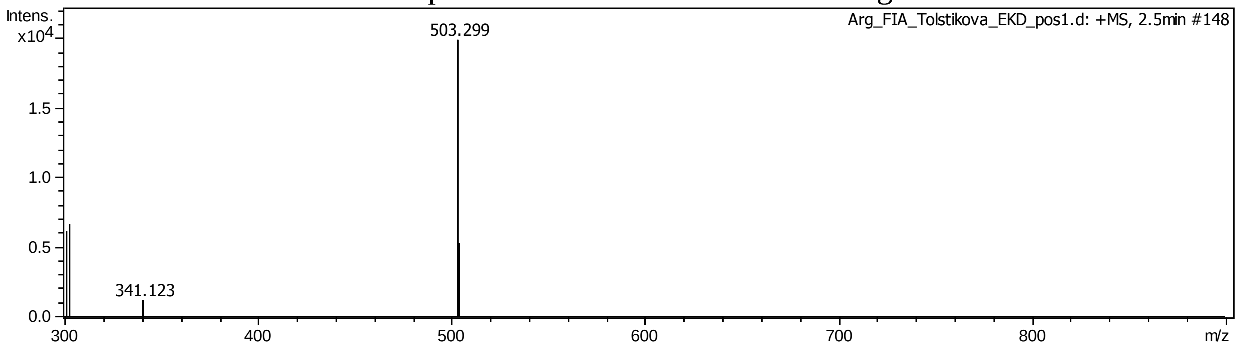

We see that the positive ions have no ion with m/z=481 ($(M+H)^+$), there is a peak with m/z=503 - this is the peak of the cation $(M+Na)^+$ and, additionally, there are isotopic peaks of the cluster.

Mass spectrum in the region of 505 daltons:

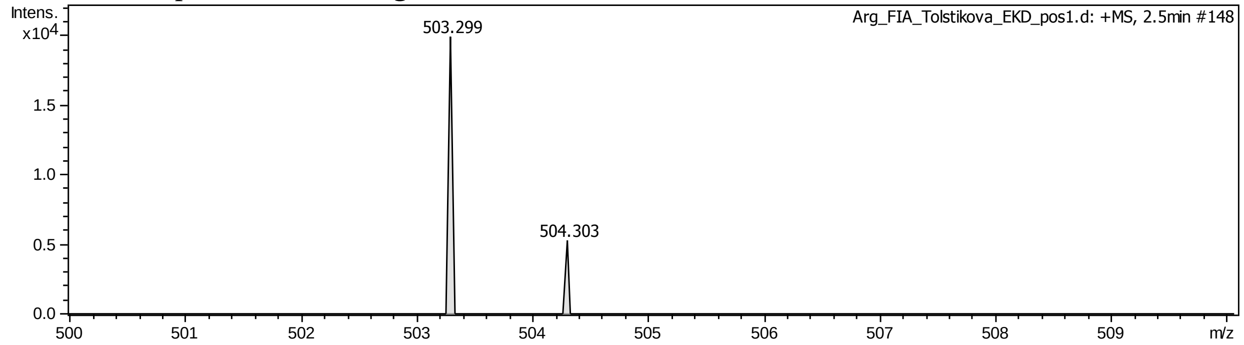

Simulation with $C_{27}H_{44}O_7Na^+$, m/z=503, recorded mass spectrum above, simulation below:

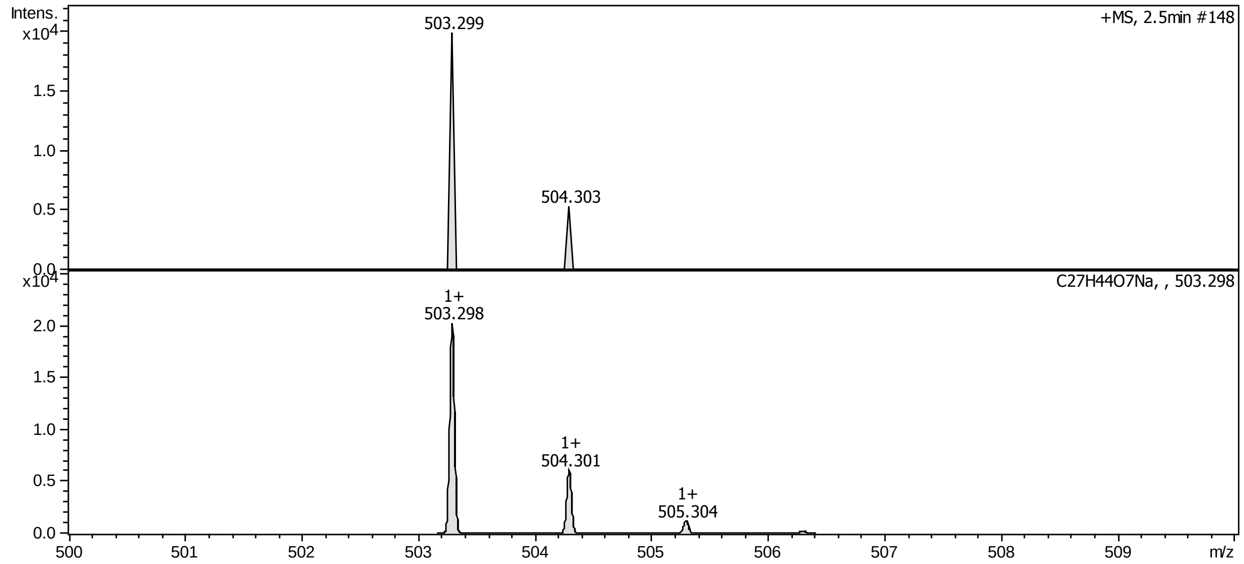

Thus: with an error of about 0.001 daltons (2.0 m.d.) this is the structure, according to the isotopic distribution also looks like it.

2.2. Leuzea extract sample

Mass spectra were recorded on a solution of 1 mg/cm3 of the sample in ethyl alcohol, direct input mass spectrometry (without chromatographic separation) on a Bruker mi-crOTOFQ instrument, electrospray ionization (ESI) method, negative ion registration mode, calibration mixture - arginine solution in acidified formic acid with bidistilled water.

General view of the mass spectrum in the 300-900 daltons range:

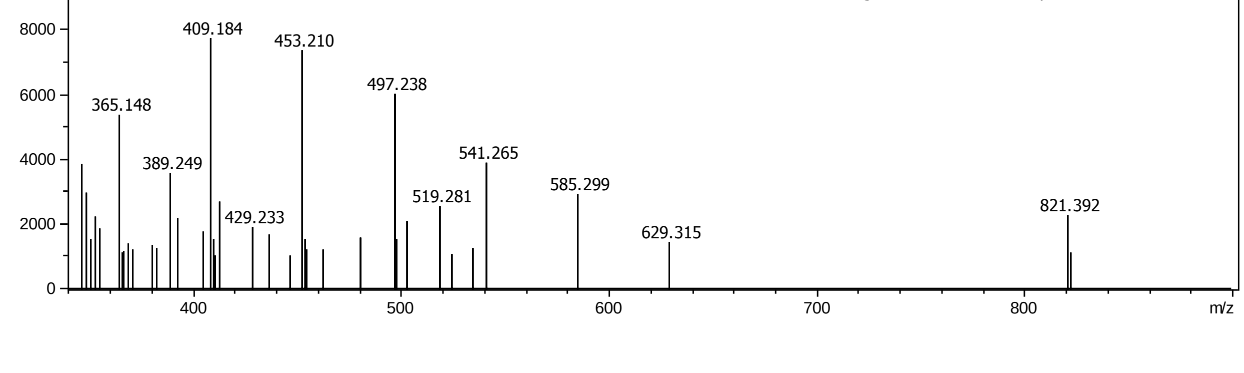

It can be seen that there is no ion with m/z=481 ($(M+H)^+$) in positive ions, there is a peak with m/z=503 which is the peak of cation $(M+Na)^+$. But the substance in the sample is small - the intensity of the line is very low.

The mass spectrum is around 505 daltons:

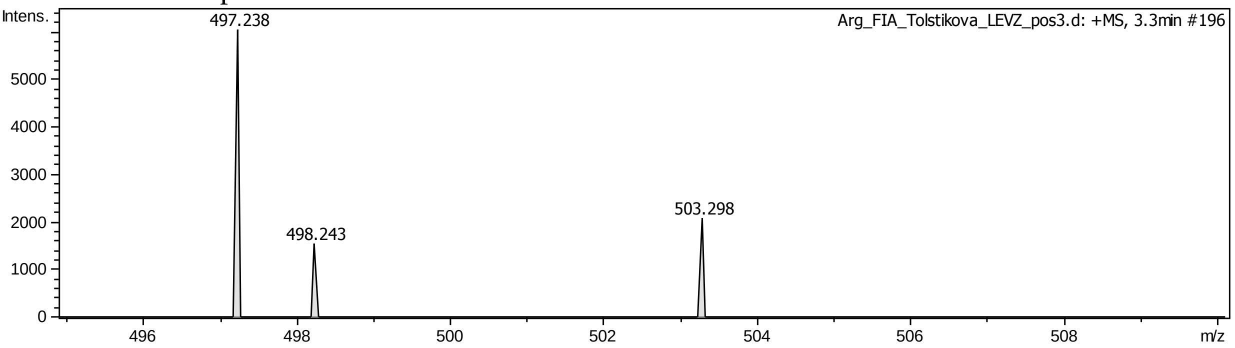

Simulation with $C_{27}H_{44}O_7Na^+$, m/z=503, recorded mass spectrum above, simulation below:

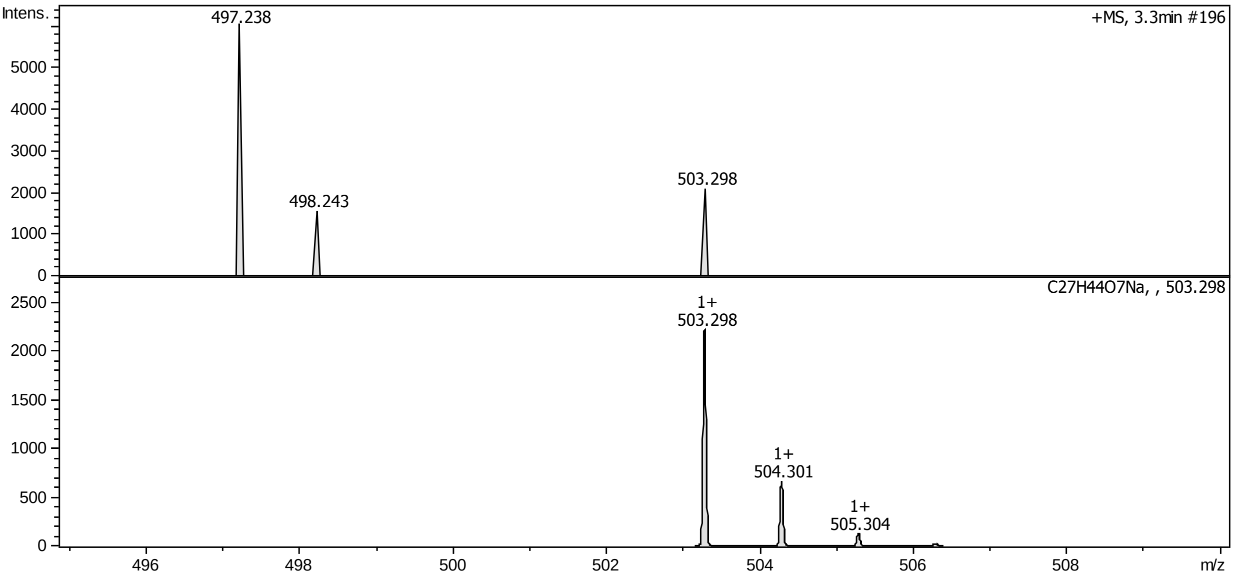

Thus: with an error of less than 0.001 daltons (less than 2.0 m.d.) this is that structure.
