# Supplementary material for: Effect of the Composition of Leuzea and Cranberry Meal Extracts on Metabolic Processes in Norm and Pathology
Source: Pharmaceuticals (Basel). 2023 May 19;16(5):768. doi: 10.3390/ph16050768 (PMC10221024; doi:10.3390/ph16050768)
Supplement: Supplementary file 1 [file pharmaceuticals-16-00768-s001.zip › File S3.pdf]

Экстракты Алтая
Только природные компоненты

Legal address: Altai Extracts LLC, Russia, Altai Territory, 656011, Barnaul, 24a/1 Kalinina Ave.
Production address: Altai Extracts Ltd., Russia, Altai Territory, 656011, Barnaul, 24A/1 Kalinina Ave., tel. +7 (983) 175-41-10

PASPORT № 0510 of 27.05.2020

**Product Name:**	Leuzea extract (root)	**Botanical Source**	Rhaponticum carthamoides
**Batch No.**	475	**Extractor**	Water
**Batch Quantity, kg**	23,49	**Manufacture Date:**	March 2020

Item	Specification	Method	Test result
Appearance	Amorphous powder or granulate of light green or brown to dark color. Hygroscopic. Allowed caking (clumping).	Organoleptic	Conforms
Odor & Taste	Characteristic	Organoleptic	Conforms
Humidity, %*	NMT 8,0	GOST 24027.2-80	4,6
Grinding degree	NMT 0,5 mm	GOST 26573.3	Conforms
Ecdystene content, %*	NLT 0,3	M 4.1.1672-03	0,3
**Microbiological Control**			
E.coli	Not allowed in 1 g	GOST 30726-2001	Conforms
S.aureus	Not allowed in 0,1 g	GOST 31746-2012	Conforms
Coliform bacteria (Klebsiella, Enterobacter, Citrobacter, and Hafnia)	Not allowed in 0,1 g	GOST 31747-2012	Conforms
Yeast & Mold, cfu/g	$1x10^2$	GOST 10444.12-2013	Conforms
Number of mesophilic aerobic and facultatively anaerobic microorganisms, cfu/g	$5x10^4$	GOST 10444.15-94, ISO 7218-2011	Conforms
Salmonella	Not allowed in 10	GOST 31659-2012	Conforms
Lead<br>Arsenic<br>Cadmium<br>Mercury	NMT 5,0<br>NMT 3,0<br>NMT 1,0<br>NMT 1,0	GOST 31671-2012<br>GOST 31671-2012<br>GOST 31671-2012<br>GOST M 54639-2011	Conforms<br>Conforms<br>Conforms<br>Conforms

Storage	In dry, well-ventilated and protected from direct sunlight rooms at a temperature not exceeding + 25 °C and relative humidity not exceeding 75%.
Shelf Life	3 years under storage conditions

Conclusion: Batch No.475 complies with the requirements of TS 9199-002-10015296-2014, mod 1, TR TS 021/2011 "On the safety of food products," TR TS 022/2011 "Food products with respect to its labeling" on the indicators tested.

Note (*): Tests were conducted by a certified laboratory LLC "AIC TEST" (Biysk, №RA.RU.21AYU83), protocol of laboratory tests №1239 from 27.05.2020.

Зам.директора по качеству ООО «Экстракты Алтая» А.П. Нестеров
