# Supplementary material for: Effect of the Composition of Leuzea and Cranberry Meal Extracts on Metabolic Processes in Norm and Pathology
Source: Pharmaceuticals (Basel). 2023 May 19;16(5):768. doi: 10.3390/ph16050768 (PMC10221024; doi:10.3390/ph16050768)
Supplement: Supplementary file 1 [file pharmaceuticals-16-00768-s001.zip › File S4.pdf]

МИНОБРНАУКИ РОССИИ
Федеральное государственное бюджетное учреждение науки
Новосибирский институт органической химии им. Н.Н. Ворожцова
Сибирского отделения Российской академии наук
просп. Академика Лаврентьева, д. 9, г. Новосибирск, 630090, Российская Федерация
Тел. (383) 330-88-50, Факс: (383) 330-97-52 E-mail: benzol@nioch.nsc.ru
ОКПО 03533903, ОГРН 1025403651921, ИНН/КПП 5408100191/540801001

PASSPORT
CRANBERRY TRITERPENE ACID EXTRACT

Batch No. 03-21 Lab. No. 2065

Manufacture Date: May 2021 Mass: 100 г. Number of seats: 1

Regulatory document: ВФС 42-3616-00

**Item**	**Test result**
1. Appearance	Yellow powder
2. Mass fraction of volatiles, %	3,3
3. Triterpene acids content (HPLC) converted to ursolic acid, %	40
4. Mass fraction of ash residue, %	0,2

Microbiological Control *

Item	Units of measure	Test result	Specification	Method
Number of mesophilic aerobic and facultatively anaerobic microorganisms	cfu/g	Less $5x10^4$	NMT $5x10^4$	GOST 10444.15
Coliform bacteria (Klebsiella, Enterobacter, Citrobacter, and Hafnia)	-	Not detected in 0,1 g	Not allowed in 0,1 g	GOST 31747
E.coli	-	Not detected in 1,0 g	Not allowed in 0,1 g	GOST 30762
Salmonella	-	Not detected in 25 g	Not allowed in 25 g	GOST 31659-(ИСО 6579:2002)
Yeast & Mold	cfu/g	Less 10	NMT 100	GOST 10444.12

Note (*): Microbiological control was carried out by the Test Laboratory of Biotechnological Control of Research Center Research and Production Firm LLC. Test protocol №83578505 of 18.05.2021.

Shelf life: 2 years if the storage conditions are observed

Storage: In dry, well-ventilated and protected from direct sunlight rooms at a temperature not exceeding + 25 °C and relative humidity not exceeding 75%.

Conclusion: Batch No.03-21 complies with the requirements of TS 9199-002-10015296-2014 "About food safety", TR TS 022/2011 "Food products and part of its labelling" according to the checked parameters.
